# Temporal dynamics in total excess mortality and COVID-19 deaths in Italian cities

**DOI:** 10.1186/s12889-020-09335-8

**Published:** 2020-08-27

**Authors:** Paola Michelozzi, Francesca de’Donato, Matteo Scortichini, Patrizio Pezzotti, Massimo Stafoggia, Manuela De Sario, Giuseppe Costa, Fiammetta Noccioli, Flavia Riccardo, Antonino Bella, Moreno Demaria, Pasqualino Rossi, Silvio Brusaferro, Giovanni Rezza, Marina Davoli

**Affiliations:** 1 Department of Epidemiology, Lazio Regional Health Service, ASL Roma 1, via Cristoforo Colombo, 112, 00147 Rome, Italy; 2 National Health Institute, Viale Regina Elena, 299, 00161 Rome, Italy; 3 Epidemiology Unit, ASL TO3, Via Sabaudia 164, 10095 Grugliasco, TO Italy; 4 Health Prevention Directorate, Italian Ministry of Health, via Giorgio Ribotta, 5, 00144 Rome, Italy

**Keywords:** Surveillance system, Total excess mortality, COVID-19-related death, Mortality displacement, Demographic factors

## Abstract

**Background:**

Standardized mortality surveillance data, capable of detecting variations in total mortality at population level and not only among the infected, provide an unbiased insight into the impact of epidemics, like COVID-19 (Coronavirus disease). We analysed the temporal trend in total excess mortality and deaths among positive cases of SARS-CoV-2 by geographical area (north and centre-south), age and sex, taking into account the deficit in mortality in previous months.

**Methods:**

Data from the Italian rapid mortality surveillance system was used to quantify excess deaths during the epidemic, to estimate the mortality deficit during the previous months and to compare total excess mortality with deaths among positive cases of SARS-CoV-2. Data were stratified by geographical area (north vs centre and south), age and sex.

**Results:**

COVID-19 had a greater impact in northern Italian cities among subjects aged 75–84 and 85+ years. COVID-19 deaths accounted for half of total excess mortality in both areas, with differences by age: almost all excess deaths were from COVID-19 among adults, while among the elderly only one third of the excess was coded as COVID-19. When taking into account the mortality deficit in the pre-pandemic period, different trends were observed by area: all excess mortality during COVID-19 was explained by deficit mortality in the centre and south, while only a 16% overlap was estimated in northern cities, with quotas decreasing by age, from 67% in the 15–64 years old to 1% only among subjects 85+ years old.

**Conclusions:**

An underestimation of COVID-19 deaths is particularly evident among the elderly. When quantifying the burden in mortality related to COVID-19, it is important to consider seasonal dynamics in mortality. Surveillance data provide an impartial indicator for monitoring the following phases of the epidemic, and may help in the evaluation of mitigation measures adopted.

## Background

The first COVID-19 cases in China were reported in December 2019, spreading quickly first to neighbouring countries like Japan and South Korea and, then across Europe, including Italy. On the 13th of July, the WHO declared the COVID-19 outbreak a pandemic currently affecting 210 countries and a death toll of almost 600,000 deaths and around 13 million cases [1]. A recent estimate suggested the burden can reach 1.0 to 2.4 billion people with severe COVID-19 cases, among those more vulnerable due to underlying clinical conditions [2].

The COVID-19 epidemic has been unique from several points of view in Italy [3], starting well before other European countries, with over 200,000 cases reported and one of the highest fatality rates worldwide (from 0·3% in the 30–39 age group to 20·2% in the 80+ age group) and with males at greater risk [4, 5]. From the first stages of the epidemic, the burden on hospitals and intensive care units was significant [6–8]. Moreover, the epidemic had a heterogeneous geographical distribution, with northern Italy being hit hardest with 90% of the COVID-19 death toll [9].

Standardized mortality data from rapid surveillance systems, capable of detecting variations in total mortality at population level and not only among the infected, can provide an unbiased and independent insight into the impact of epidemics like COVID-19 [10–12]. At the European level, the EuroMOMO surveillance network which includes 24 countries (including Italy) and is part of ECDC surveillance, estimated an overall excess from week 1 to 18 of 2020 of 185,287 deaths thus higher than the official COVID-19 estimates [13].

Since the beginning of the COVID-19 outbreak, data from the Italian rapid mortality surveillance system (SiSMG), has been used to estimate the impact on mortality in near-real time in terms of excess deaths, and results have been published in weekly reports and publications [14, 15]. To date, a comparison of total mortality and COVID-19 deaths has not been carried out in Italy and can help quantify the overall burden and evaluate the direct and indirect effects of the epidemic.

SiSMG reports have shown a greater excess in mortality among the elderly (+ 49% in 15–64 age group to + 103% in the 85+ in northern cities) and among males [5] as shown in previous studies on COVID-19 deaths in Italy. Recent patient cohort studies confirm a higher infection and mortality risk among males and in the elderly [16, 17].

When quantifying the overall burden, the seasonal dynamics in mortality and the role of concurrent risk factors that influence mortality trends, especially among the pool of frail elderly with a compromised health conditions and short life span [10, 18] that are at greater risk, need to be considered. Specifically, the potential role of below-average mortality in months prior to the COVID-19 outbreak, possibly associated to the moderate influenza intensity and above average winter temperatures, need to be accounted for when estimating excess mortality.

## Methods

The paper aims to analyse the temporal trend in total excess mortality and COVID-19 deaths defined as deaths among subjects with microbiologically confirmed SARS-CoV-2 reported to the Italian National Surveillance system [19]. Furthermore, the study estimates the burden of total mortality taking into account the potential role of the mortality deficit in months prior the outbreak, by geographical area (north and centre and south), age and sex.

### SiSMG mortality data

Since 2004, an ad-hoc rapid mortality surveillance system (SiSMG) for the real-time monitoring of daily deaths has been operational in major Italian cities and allows for the routinely evaluation of the health impact of extreme weather events and influenza epidemics [10, 20]. SISMG data was provided by DEPLAZIO who manages the system on behalf of the Ministry of Health [14, 21]. Individual records include: date of birth and death, sex, place of death (in-hospital, out-of-hospital), and municipalities of birth, residency and death. SiSMG data includes deaths occurring among residents dying in the municipality of residency. The study period was comprised between 1/12/2020 and 20/04/2020.

### COVID-19 deaths data

Data on COVID-19 deaths was provided by the Italian National Health Institute (ISS) that coordinates the Italian National Integrated COVID-19 Surveillance System that collects laboratory RT-PCR confirmed cases of SARS-CoV-2 infection in Italy through an online secure database [19]. A COVID-19 related death is defined as any death with a laboratory confirmed SARS-CoV-2 infection, regardless of pre-existing disease. Each record also reports information on the municipality of residence, region of death, sex, and age. Data is published in near real-time and epidemiological reports are provided weekly. Data was available for the same period as SiSMG data.

### Statistical methods

The 31 Italian cities included in SiSMG, corresponding to 53.8% (10,593,915 residents) of urbanized Italian areas, were grouped in into two geographical areas: north (12 cities) and centre and south (19 cities). The two areas had extremely different patterns of COVID-19 onset times, absolute numbers of cases, velocity of infection spread and mortality toll, therefore were analysed separately. Daily baseline mortality was computed as the average number of deaths registered in the same week and weekday of the previous five years for each area, age group and sex. Excess daily mortality was calculated as the difference between observed and baseline mortality counts. We then split the time-series into a pre-COVID and a COVID period starting from the date in which consecutive COVID-19 deaths were recorded (north: 29th of February, centre and south: 11th of March). For the COVID period, we calculated total excess mortality and compared it to the number of COVID-19 deaths. Residual deaths were derived by difference.

Deficit mortality in the pre-COVID period was quantified to have a measure of the potential mortality displacement in the pool of susceptible individuals which were then exposed to the virus. Finally, we computed the ratio between the deficit mortality in the pre-COVID period and the total excess in the COVID period as an estimate of the mortality displacement phenomenon.

All analyses were conducted using R statistical software (version 3.6.0) [22].

## Results

**Fig. 1:**
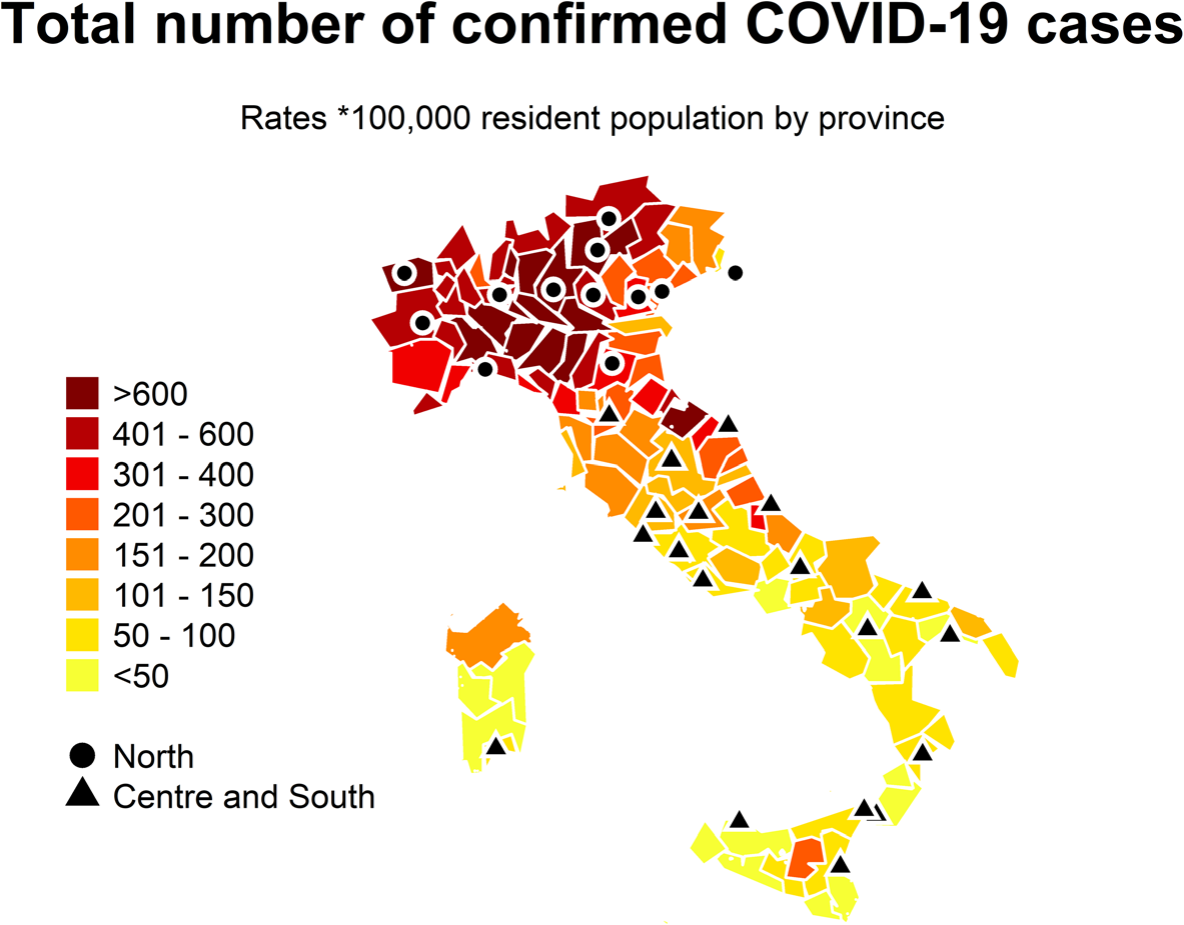
Distribution of COVID-19 confirmed cases by province (rates by 100,000 resident population, data up to May 4th) and location of the Italian cities included in the study (The map has been generated using R statistical software and the source of the shape file is the Italian Institute of Statistics released with Creative Commons License – Attribution – 3.0)

The map in Fig. 1 shows the location of the 31 Italian cities and COVID-19 case rates (per 100,000 residents) by province [23], showing a clear north-south divide in the impact of the virus.

**Fig. 2:**
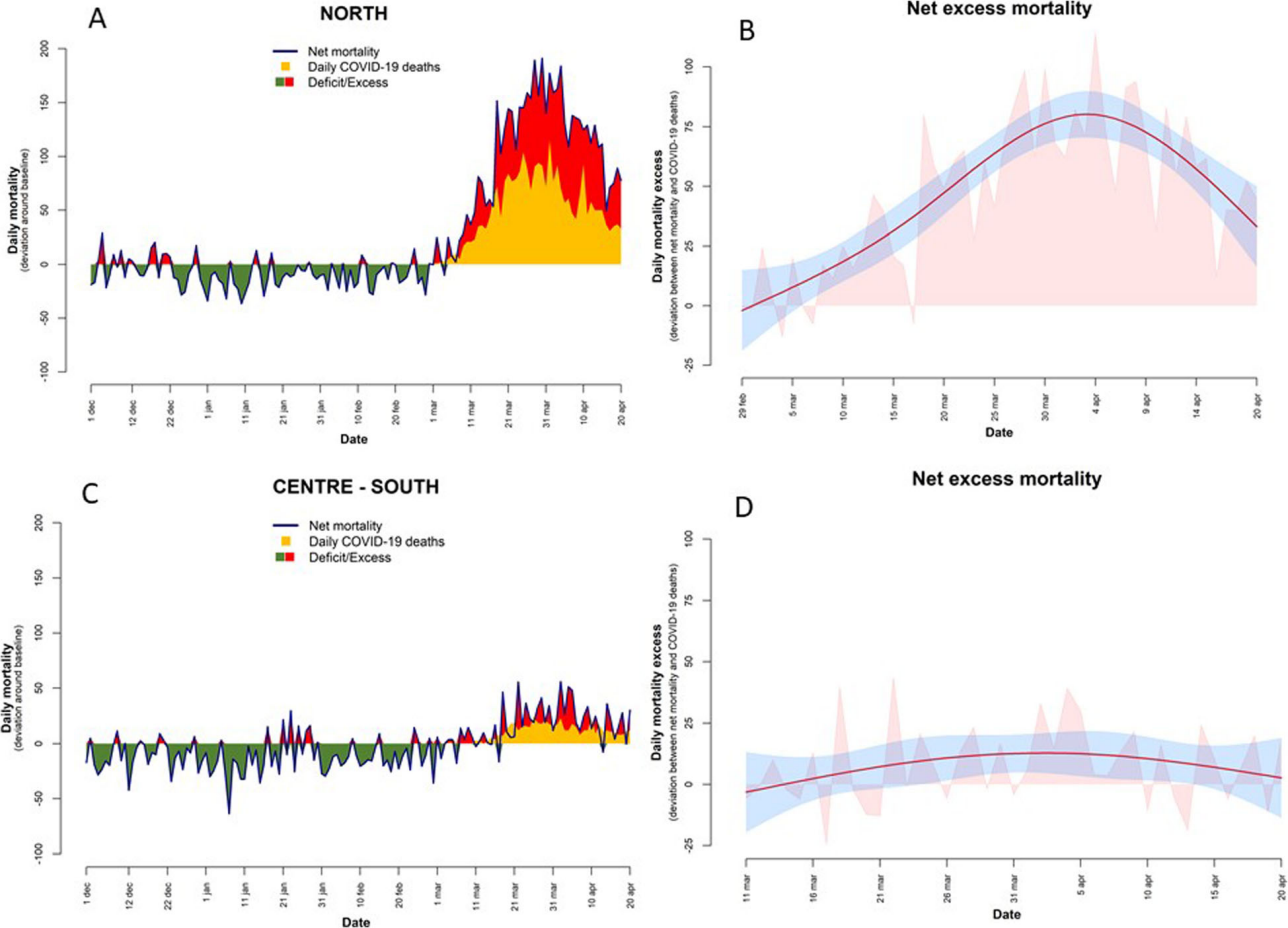
Mortality trends^a^ before and during the COVID-19 outbreak by geographical area^b^. Footnotes. ^a^ Panel **a** and **c** show daily deviations between observed and baseline mortality, together with COVID-19 daily deaths. Panel **b** and **d** present daily trends in net excess mortality (e.g. total mortality after removal of baseline and COVID-19 deaths) with parametric curve derived from natural spline with 3 degrees of freedom (red line) and 95% confidence bands (light blue shaded area) for Northern and Central and Southern Italy respectively. ^b^ Panel **a** and **b**: northern Italy (12 cities); panel **c** and **d**: central and southern Italy (19 cities)

Figure 2 shows a consistent deficit in mortality in the pre-COVID period (green area) in both geographical areas. In northern cities total mortality increased abruptly from the beginning of March, reaching a peak at the end of the month, mirrored by the trend in COVID-19 deaths but not in absolute numbers. The plot on the right shows the temporal trend in the proportion of excess deaths not reported as COVID-19 deaths. At the beginning, when total numbers were low, the residual component was limited, while it reached its maximum in correspondence to the peak of the epidemic and greatest excess in mortality. In central and southern Italy, total excess mortality due to COVID-19 started later on and was more contained, and the gap between total excess mortality and COVID-19 deaths was limited with no clear trend.

**Fig. 3:**
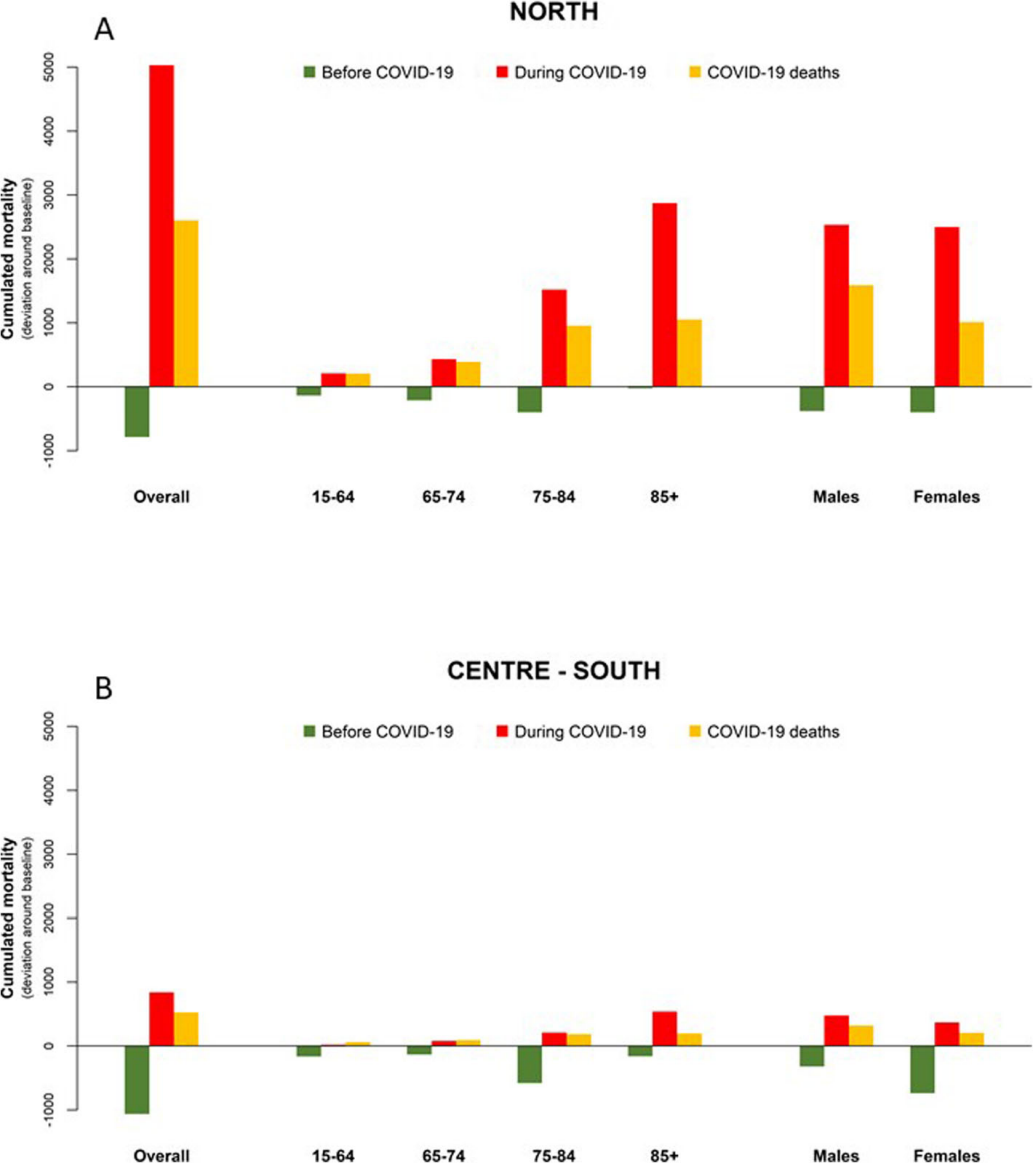
Cumulative mortality differences^a^ before and during COVID-19 outbreak, by geographical area^b^, age and sex. Footnotes. ^a^ Green and red bars represent cumulative differences between observed and expected deaths before and during the COVID-19 outbreak, respectively; yellow bars show cumulative COVID-19 deaths. ^b^ Panel **a**: northern Italy (12 cities); panel **b**: central and southern Italy (19 cities)

Total excess mortality and COVID-19 deaths by age and sex, for the two periods, are shown in Fig. 3 and Table 1. In the north of Italy, excess mortality in the COVID-19 period (red bars) was greater than COVID-19 mortality (yellow bars): out of a total of 5028 excess deaths, only 52% were coded as COVID-19. The excess was only marginally explained by the deficit in mortality in the pre-epidemic period (green bars, Fig. 3). The harvesting fraction, quantified as the portion of the excess needed to fill the gap in mortality occurred during the pre-COVID period, was, of 16% overall (Table 1).

**Table 1:** Quota of excess mortality from COVID-19 deaths and from displaced deaths, by geographical area^a^, age and sex

	Total excess mortality during COVID-19^b^	COVID-19 deaths	% COVID-19^c^	Deficit mortality before COVID-19^d^	% displaced mortality^e^
**NORTHERN ITALY** (COVID-19 period starts on February 29th, 2020)					
**Overall**	5028	2601	52%	− 787	16%
Age (years)					
15–64	210	205	98%	− 141	67%
65–74	429	388	90%	−214	50%
75–84	1522	957	63%	− 404	27%
85+	2871	1051	37%	−22	1%
Sex					
Males	2532	1590	63%	− 383	15%
Females	2496	1011	41%	−404	16%
**CENTRAL and SOUTHERN ITALY** (COVID-19 period starts on March 11th, 2020)					
**Overall**	839	522	62%	-1063	> 100%
Age (years)					
15–64	19	59	> 100%	− 168	> 100%
65–74	79	87	> 100%	−137	> 100%
75–84	207	182	88%	− 578	> 100%
85+	539	194	36%	−164	31%
Sex					
Males	474	318	67%	− 324	68%
Females	365	204	56%	− 738	> 100%

^a^ northern Italy (top): 12 cities; central and southern Italy (bottom): 19 cities

^b^ Calculated as the difference between observed mortality and baseline in the COVID-19 period

^c^ Calculated as the ratio between COVID-19 deaths and total excess mortality in the COVID-19 period

^d^ Calculated as the difference between observed mortality and baseline in the period before COVID-19

^e^ Calculated as the ratio between deficit mortality in the period before COVID-19 and total excess mortality during COVID-19

Results by age showed very diverse patterns, with COVID-19 deaths explaining almost all the excess among the youngest groups and only a minor portion among the very old (aged 85+ years). Conversely, the proportion of excess explained by the mortality deficit in the pre-COVID period rapidly declined from 67% in the youngest to 1% to the oldest age group. Results by sex showed a negligible displacement in both men and women (16% of the total excess mortality), with a higher fraction of excess explained by COVID-19 deaths among males (63%).

In central and southern cities, deficit mortality in the pre-COVID period was greater, and excess mortality during the epidemic was more contained (Fig. 3). Overall, COVID-19 deaths accounted for 62% of the total excess mortality, with similar differences by age group as in northern cities. The mortality deficit in the pre-COVID period was greater than the excess mortality registered during the outbreak among those aged below 85 years and among females, resulting in proportions of potential displacement greater than 100% in these subgroups (Table 1).

## Discussion

Our study provides a useful insight on the impact of COVID-19 in Italy both in terms of total excess mortality and the proportion of COVID-19 deaths. Standardized total mortality surveillance data and laboratory confirmed COVID-19 cases are important tools for public health, not only for monitoring the impact but also for a more efficient response of health services towards those most at risk. Moreover, total mortality data paralleled by COVID-19 laboratory confirmed deaths can provide a more detailed picture of infection severity on a population, and the possible indirect effects (e.g. due to containment measures). Results confirm that cities in the North of Italy had the greatest mortality burden and only half of the excess deaths were reported as COVID-19 deaths. Moreover, a consistent difference by age group was observed: in the youngest groups (15–64 and 65–74 years) COVID-19 deaths account for all the excess, whereas among subjects aged 85+ years only 37% of excess deaths were reported as COVID-19 deaths. It should be noted that in the younger age groups the excess is mostly among subjects aged 50 years or more, as reported in other studies [4, 5].

The mismatch between excess mortality and COVID-19 reported deaths may be attributed to differential testing strategies and to hospital and health system overload especially during the epidemic peak as documented in previous studies both from Italy [24] and other countries [12, 25]. Different aspects need to be taken into account when interpreting the difference in the portion of the excess explained by COVID-19 deaths by age group. In fact, at the beginning of the epidemic, the test was recommended for patients with more severe clinical symptoms related to COVID-19 and required hospitalization, while it was uncommon among subject with mild symptoms, or symptoms not directly attributable to COVID-19. This could be the case for the elderly population due to the difficulties of recognizing COVID-19-like symptoms or even to a non-regulated discrimination in favour of the youngest for the limited testing offer. Furthermore, among those residing in nursing homes, testing was possibly carried out with some delay when the epidemic had already spread in the closed setting of high-risk patients. In the province of Bergamo in the Lombardy region, more than 600 elderly residing in nursing homes died between the 7th and the 27th of March, 2020 [26].

In this study, age and sex were confirmed as risk factors for COVID-related mortality. The elderly (aged 65+ years) and males show a higher mortality, confirming previous findings [5, 16, 27–29]. A possible explanation of the excess in mortality in the oldest age group can be due to multiple comorbidities which rapidly deteriorate with virus infection and cannot be distinguished from COVID-19 clinical signs. Moreover, the excess in males could be related to a greater susceptibility to viruses related to male hormones [17, 28]. In our study, the overlap between total and COVID-19 related mortality was greater among males, suggesting a more severe clinical progression requiring hospitalization, as suggested by the pro-inflammatory pathway activated in old men by the reduced testosterone levels [16, 28].

In parallel, the COVID-19 epidemic produced an unprecedented mobilization of the health system to respond to this emergency, thus reducing elective treatments in all settings. While on the other hand, the use of emergency services saw a dramatic decline during the COVID-19 outbreak, suggesting a collective fear of being infected within a hospital setting. A study conducted in the Lazio region, Italy showed a drop in emergency room visits during the COVID-19 epidemic, with a 50% reduction in acute coronary syndrome and acute cerebrovascular disease with a possible impact on mortality [30]. Similarly, a US study showed a reduction in emergency room visits during the containment period suggesting that underlying determinants are not affected by population characteristics [31]. Interestingly, the study also found a larger reduction in myocardial infarction among males and in the 65–74 age group, providing another potential explanation of the observed heterogeneity in the proportion of COVID-19 deaths by age and gender [31]. An increase in out-of-hospital cardiac arrests was also reported in the Lombardy Region during COVID-19 outbreak [32]. Other studies suggest a reduction in cancer screening rates during lockdown periods in some countries [33]. Maintaining continuity of essential services and empowerment of citizens is essential to protect the population especially elderly, with chronic diseases.

Furthermore, our study provides estimates of total excess mortality taking into account mortality deficits in previous months. Below average mortality was observed mostly in the 75–84 age group in previous months and was greater in central and southern cities. It is interesting to note is that the mortality deficit during the COVID-19 epidemic was exceeded in the northern regions, while in central and southern cities, when taking into account the mortality deficit of previous months, no excess was observed. Among the very old (85+ years) there was hardly any displacement in mortality as one would expect among this frail population. Results show that when quantifying the mortality burden related to COVID-19, it is important to consider seasonal dynamics in mortality and possible deficits in previous months.

The Italian Government issued national lockdown on the 9th of March, with an escalation of mitigation measures such as staying at home, closure of schools, shutting down of non-essential businesses such as restaurants and bars, and the transition to smart working where possible across public and private sectors [34]. From the 4th of May measures were relaxed across Italy and we entered the re-opening phase. Epidemic models suggest that mitigation measures in Italy, probably had a key role in reducing the impact in central and southern regions compared to the north suggesting their potential effectiveness in containing the epidemic [35, 36].

Whether the new coronavirus is similar or not to other viruses of the same family is still unclear. Other coronaviruses had a strong seasonality with a winter peak and very limited virus circulation in the May to November period [37]. Some scenarios have been hypothesized for the coming months, one possibility is that the higher temperature of the coming months could reduce virus transmission as suggested by a few studies [38].

## Conclusions

Our results underline the importance of considering both total excess mortality and COVID-related death, the temporal trends and the potential displacement in mortality of the pre-pandemic period when assessing the impact of the epidemic in order to account for indirect effects and the potential under notification of cases due to testing policies put in place. Data from SiSMG and the National COVID-19 Surveillance System are valuable tools for the monitoring of the following phases of the epidemic, and will allow the evaluation of mitigation measures adopted at both local and national level.

## Data Availability

The data that support the findings of this study are available from the Ministry of Health and the National Health Institute but restrictions apply to the availability of these data, which were used under license for the current study, and so are not publicly available. Data are however available from the authors upon reasonable request and with permission of the two public authorities.

## Abbreviations

COVID-19: Coronavirus Disease
SARS-CoV-2: Severe acute respiratory syndrome coronavirus 2
SiSMG: Italian rapid mortality surveillance system
RT-PCR: Reverse transcription polymerase chain reaction
