# Publisher Correction to: Temporal dynamics in total excess mortality and COVID-19 deaths in Italian cities

**DOI:** 10.1186/s12889-020-09398-7

**Published:** 2020-08-31

**Authors:** Paola Michelozzi, Francesca de’Donato, Matteo Scortichini, Patrizio Pezzotti, Massimo Stafoggia, Manuela De Sario, Giuseppe Costa, Fiammetta Noccioli, Flavia Riccardo, Antonino Bella, Moreno Demaria, Pasqualino Rossi, Silvio Brusaferro, Giovanni Rezza, Marina Davoli

**Affiliations:** 1 Department of Epidemiology, Lazio Regional Health Service, ASL Roma 1, via Cristoforo Colombo, 112, 00147 Rome, Italy; 2 National Health Institute, Viale Regina Elena, 299, 00161 Rome, Italy; 3 Epidemiology Unit, ASL TO3, Via Sabaudia 164, 10095 Grugliasco, TO Italy; 4 Health Prevention Directorate, Italian Ministry of Health, via Giorgio Ribotta, 5, 00144 Rome, Italy

**Correction to: BMC Public Health 20, 1238 (2020)**

**https://doi.org/10.1186/s12889-020-09335-8**

In the publication of the original article [1], several modifications were not implemented. These changes related to updated reference information and textual clarifications. In this correction article the significant changes are provided in Supplementary file 1.These changes do not impact the conclusion of the article. The original article has been updated.

## Supplementary information

**Additional file 1.**

## References

[1] Michelozzi P, de’Donato F, Scortichini M (2020). Temporal dynamics in total excess mortality and COVID-19 deaths in Italian cities. BMC Public Health.

