# Supplementary material for: Publisher Correction to: Temporal dynamics in total excess mortality and COVID-19 deaths in Italian cities
Source: BMC Public Health. 2020 Aug 31;20:1325. doi: 10.1186/s12889-020-09398-7 (PMC7457443; doi:10.1186/s12889-020-09398-7)
Supplement: Supplementary file 1 — Additional file 1. [file 12889_2020_9398_MOESM1_ESM.docx]

| **Incorrect** | **Correct** |
| --- | --- |
| Surveillance data provides an impartial indicator for monitoring the following phases of the epidemic, and may help in the evaluation of mitigation measures adopted. | Surveillance data provide an impartial indicator for monitoring the following phases of the epidemic, and may help in the evaluation of mitigation measures adopted. |
| On the 11th of May, the WHO declared the COVID-19 outbreak a pandemic currently affecting 210 countries and a death toll of more than 550,000 deaths and almost 13 million cases [1]. | On the 13^th^ of July, the WHO declared the COVID-19 outbreak a pandemic currently affecting 210 countries and a death toll of almost 600,000 deaths and around 13 million cases [1]. |
| SiSMG reports have shown a greater excess in mortality among the elderly (+ 49% in 15–64 age group to + 103% in the 85+ in northern cities) and among males [5]. | SiSMG reports have shown a greater excess in mortality among the elderly (+ 49% in 15–64 age group to + 103% in the 85+ in northern cities) and among males as shown in previous studies on COVID-19 deaths in Italy [5]. |
| Mortality trends^a^ before and during the COVID-19 outbreak by geographical area^b^. Footnotes. ^a^ Panel **a** and **c** show daily deviations between observed and baseline mortality, together with COVID-19 daily deaths for Northern and Central and Southern Italy respectively. | Mortality trends^a^ before and during the COVID-19 outbreak by geographical area^b^. Footnotes. ^a^ Panel **a** and **c** show daily deviations between observed and baseline mortality, together with COVID-19 daily deaths. |
| The mortality deficit in the pre-COVID period were greater… | The mortality deficit in the pre-COVID period was greater… |
| It should be noted that in the younger age group… | It should be noted that in the younger age groups… |
| Different aspects need to be taken into account when interpreting the difference of COVID-19 deaths by age group. | Different aspects need to be taken into account when interpreting the difference in the portion of the excess explained by COVID-19 deaths by age group. |
| An increase in out-of-hospital cardiac arrests was also reported in the Lombardy Regional | An increase in out-of-hospital cardiac arrests was also reported in the Lombardy Region |
| Epidemic models suggest that mitigation measures, similar to those put in place in Italy, may have a key role in reducing the impact in central and southern regions compared to the north suggesting their potential effectiveness in containing the epidemic [[35](https://bmcpublichealth.biomedcentral.com/articles/10.1186/s12889-020-09335-8#ref-CR35), [36](https://bmcpublichealth.biomedcentral.com/articles/10.1186/s12889-020-09335-8#ref-CR36)]. | Epidemic models suggest that mitigation measures in Italy, probably had a key role in reducing the impact in central and southern regions compared to the north suggesting their potential effectiveness in containing the epidemic [[35](https://bmcpublichealth.biomedcentral.com/articles/10.1186/s12889-020-09335-8#ref-CR35), [36](https://bmcpublichealth.biomedcentral.com/articles/10.1186/s12889-020-09335-8#ref-CR36)]. |
| PP provided data from the COVID surveillance system and revised the manuscript. | PP provided data from the COVID-19 surveillance system and revised the manuscript. |
